# Environmental Monitoring with Distributed Mesh Networks: An Overview and Practical Implementation Perspective for Urban Scenario †

**DOI:** 10.3390/s19245548

**Published:** 2019-12-16

**Authors:** Aleksandr Ometov, Sergey Bezzateev, Natalia Voloshina, Pavel Masek, Mikhail Komarov

**Affiliations:** 1 Tampere University, 33720 Tampere, Finland; aleksandr.ometov@tuni.fi; 2 Saint Petersburg State University of Aerospace Instrumentation, 190000 St. Petersburg, Russia; bsv@aanet.ru; 3 ITMO University, 197101 St. Petersburg, Russia; nvvoloshina@itmo.ru; 4 Brno University of Technology, 60190 Brno, Czech Republic; masekpavel@vutbr.cz; 5 National Research University Higher School of Economics, 101000 Moscow, Russia

**Keywords:** environmental monitoring, authentication mechanism, security, wireless sensor network, distributed systems

## Abstract

Almost inevitable climate change and increasing pollution levels around the world are the most significant drivers for the environmental monitoring evolution. Recent activities in the field of wireless sensor networks have made tremendous progress concerning conventional centralized sensor networks known for decades. However, most systems developed today still face challenges while estimating the trade-off between their flexibility and security. In this work, we provide an overview of the environmental monitoring strategies and applications. We conclude that wireless sensor networks of tomorrow would mostly have a distributed nature. Furthermore, we present the results of the developed secure distributed monitoring framework from both hardware and software perspectives. The developed mechanisms provide an ability for sensors to communicate in both infrastructure and mesh modes. The system allows each sensor node to act as a relay, which increases the system failure resistance and improves the scalability. Moreover, we employ an authentication mechanism to ensure the transparent migration of the nodes between different network segments while maintaining a high level of system security. Finally, we report on the real-life deployment results.

## 1. Introduction

To date, the development of various industries has brought a tremendous impact on our climate. According to the National Aeronautics and Space Administration (NASA), global climate change already has effects that can be observed in the environment. Glaciers have shrunk, ice on rivers and lakes is melting ahead of time, plant and animal habitats have changed, and trees bloom ahead of expected dates [1]. The previously predicted effects from global climate change are already happening: (i) loss of sea ice; (ii) accelerated sea-level rise; and (iii) more intense heat waves [2].

According to the European Commission, the main impact on climate change is due to the greenhouse effect, which is mainly caused by CO${}_{2}$ emissions, in turn being mainly a result of human activities (64% of global warming is human-made [3]). Its concentration in the atmosphere is currently 40% higher than it was during the beginning of industrialization [4]. This impact is mainly due to: (i) burning coal; (ii) gas and oil; (iii) deforestation; (iv) increasing livestock farming; and (v) a rise in fluorinated gases amount.

One of the critical aspects in reducing the negative impact on the climate is efficient monitoring of the environmental data in addition to prompt actions aiming to reduce such impact in dedicated areas. Indeed, many researchers are actively improving and developing new solutions utilized for monitoring. After broad adoption of the Internet of Things (IoT), growing interest from industry, researchers, governments, and developers was given to IoT’s particular niche — Industrial IoT (IIoT) [5]. This sector aims at covering the machine-to-machine communications (M2M) domain and topics related to modern Wireless Sensor Networks (WSNs) including ones operating in both licensed [6,7] and unlicensed bands [8,9].

IIoT provides a number of main machine-oriented development directions, including: (i) factory automation; (ii) mission-critical communications; and, generally, (iii) monitoring [10]. Historically, monitoring solutions are well-known from WSNs, and the world of today could not be imagined ignoring this section of IIoT [11].

In this context, environmental and agricultural monitoring fields are ideal candidates for trialing and deploying the IIoT solutions [12]. No doubt, the utilization of sensors may be vastly applicable for it, e.g., for monitoring of humidity, emissions, and temperature levels; for production chain control; for air pollution maps construction; for immediate alert triggers; and others.

The industrial trends of today aim at “connecting the unconnected”. Presently developed systems sometimes fall behind the expectations due to their complexity and lack of proper community support. Thus, freely programmable and advanced Cyber-Physical Systems (CPS) should replace conventional programmable logic controllers in managing physical objects [13]. Simultaneously, blind development of said systems may be harmful from the information security perspective, and threats (primarily related to authentication) should be carefully taken into consideration [14,15,16].

Current research is also vital for the analysis of technological requirements and interconnections between different characteristics for distributed ledger technology (DLT) design. Developers need to conduct a comprehensive comparison between prospective DLT designs before starting the implementation suitability for a particular application [17]. Environmental monitoring system based on mesh network approach falls into the specific domain of distributed systems, which can be implemented on the DLT basis, where sensing devices could vary depending on different manufacturers or service-providers and where the level of trust to the sensing data will be higher due to the DLT implementation. An example of a distributed mobility platform was presented in [18] demonstrating its technical feasibility and showing that the introduction of distributed mobility concept will benefit both the supply and demand sides of public transportation at the same time.

In this paper, we propose and develop the CPS system titled “Galouis”, which is a flexible environmental monitoring tool relying on the distributed network architecture. Dell-EMC carefully managed this work and supported the deployment in the metropolitan area. The main contributions of this work are:

1. Modern environmental monitoring applications and scenarios are reviewed.
2. The pairwise key-based authentication mechanism was applied for urban environmental monitoring, allowing to handle individual system operational phases, e.g., the addition of new nodes, (un-)authorized migration of the node from one network segment to another, etc.
3. An analytical framework based on Markov chain analysis that allows evaluating potential network topology changes is presented.
4. A prototype of the proposed secure distrusted sensor network (operating based on the discussed authentication mechanism) was deployed in a real-life scenario.

The paper is structured as follows. Section 2 provides an overview of the leading environmental applications and related security aspects. Section 3 provides the system description and highlights the main problematics. Section 4 overviews the developed secure operation enablers of the system. Section 5 shows the developed analytical approach and selected numerical results. Section 6 provides technical details of the prototype and real-life deployment. The last section concludes the paper.

## 2. Overview on Environmental Monitoring Applications and Main Security Specifics

**Figure 1:**
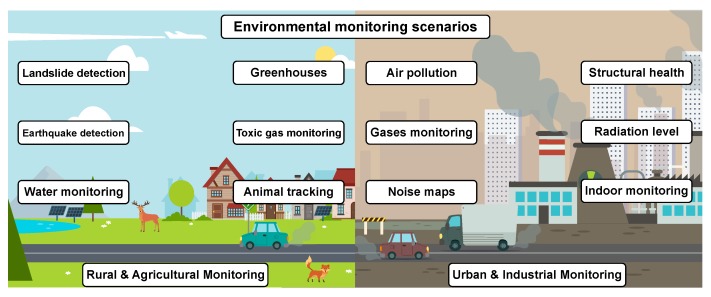
Selected monitoring scenarios and applications.

Focusing mainly on the Smart City paradigm from the IIoT perspective, the main activities of environmental monitoring could be listed as the following [19] (see selected ones in Figure 1). The first group of applications corresponds to the paradigm of urban environmental monitoring [20]. It consists of the following subgroups: (i) structural health [21]; (ii) light pollution monitoring [22]; (iii) waste management [23]; (iv) noise monitoring [24]; and (v) air pollution [25].

A massive section of this group is related to industrial control [26], aiming at: (i) indoor air quality monitoring [27], i.e., monitoring of toxic gas and oxygen levels inside chemical plants and office spaces to ensure safety; (ii) temperature monitoring [28], i.e., control of the temperature inside industrial and medical fridges with sensitive products; (iii) ozone level monitoring [29], i.e., monitoring of ozone levels inside food factories; and (iv) indoor positioning [30], i.e., indoor asset location utilizing active (ZigBee and Ultra-Wideband (UWB)) and passive (Radio Frequency Identification (RFID) and Near Field Communication (NFC)) tages. Nonetheless, security and emergency scenarios are also to be considered [31] as, for example: (i) perimeter access control [32], i.e., border surveillance and intrusion detection; (ii) dangerous liquid presence and leak detection [33,34], i.e., monitoring of the lower explosive limit of potentially dangerous gases and vapors; (iii) radiation level monitoring [35], i.e., real-time monitoring of radiation levels at nuclear facilities and surrounding areas; and (iv) explosive and hazardous gases in underground environments [36], i.e., continuous monitoring of the ambient characteristics of the mining environment.

The second big group is related to rural environmental monitoring [37]: (i) landslide and avalanche prevention [38], i.e., monitoring of soil moisture, vibrations, and earth density to detect dangerous patterns of inland conditions; (ii) earthquake early detection [39], i.e., distributed control in specific places of tremors; and (iii) forest fire detection [40], i.e., monitoring of combustion gases and preemptive fire conditions to define alert zones. A standalone section within rural monitoring is dedicated to agricultural monitoring [41] covering the following applications: (i) greenhouse parameter control [42], i.e., control of micro-climate conditions to maximize the production of fruits and vegetables and its quality; (ii) meteorological station network [43], i.e., monitoring of weather conditions in fields to forecast ice formation, rain drought, snow, or wind changes; (iii) animal tracking [44], i.e., location and identification of animals grazing in open pastures or location in big stables; (iv) wine production and quality enhancing [45], i.e., monitoring the productive cycle of high-quality wine; (v) monitoring of the toxic gas level of farm animals [46], i.e., a study of ventilation and air quality in farms and the detection of harmful gases from excrements; and (vi) compost monitoring [47], i.e., control of humidity and temperature levels in alfalfa, hay, straw, etc. to prevent fungus and other microbial contaminants.

It is important to notice that the entire deployment predictivity of the IIoT sensor network is somewhat challenging due to a significant number of nodes involved. Moreover, devices could disconnect from the network, reconnect again, or move to another segment of the network without notifying the coordinator. The use of distributed sensor networks with flexible topology requires the utilization of secure yet straightforward authentication protocols.

One of the most significant challenges of dynamic WSNs is the lack of centralized authority coordination. Such a center should provide storage, generation, and dissemination of the certificates to each sensor node operating within the public key infrastructure (PKI) paradigm [48]. If the agreement of using a single authentication center could be reached, it is relatively straightforward to perform mutual node authentication and secret key generation for secure data transmission. If there is no possibility of having just a single authentication center, a high demand to create and use reliable authentication protocols appears together with the need for the application layer management platform operating in a straightforward and flexible way.

## 3. System Description and Problem Statement

The developed system is a distributed self-organizing sensor network designed to monitor the parameters of the urban environment. It allows for the data transmission only from the trusted sensors that confirm their association with a specific network. The monitoring of the data is carried out remotely using a trusted Internet portal with a graphical user interface (GUI). A trusted addition and removal of the sensor are carried out using a smartphone application given the assumption that the device supports IEEE 802.11 protocol operation.

The system was designed considering the requirements of urban environmental services, city administration, and emergency services. In addition, some information received and processed by the system can be provided to third parties for planning mass events, as well as to citizens to inform them about the environmental situation. In the case of providing information to citizens, data may be shown in quantitative form, e.g., in the form of geographical information systems (GIS).

The developed system aims at solving the problem of promptly informing relevant services regarding possible emergency situations, allowing for better prediction and fast reaction.

The system is designed to operate in three different modes:

1. Simplex mode: The operation of the system is executed according to the “star” topology, and the transmission of messages to the Access Pontes (APs) directly using a controlled sleep mode.
2. Duplex mode: The operation of the system is executed according the mesh network mode with relaying via the closest network nodes using a controlled sleep mode.
3. Half-duplex mode: The operation the system is executed via the star topology but using a predefined sleep mode, i.e., the preset of the optimal mode for a given scenario and operating conditions are applied.

Utilization of relaying strategy in duplex mode can significantly reduce the number of APs required for a deployable wireless network reducing the system’s overall deployment cost.

The proposed system allows us to solve the task of environmental monitoring by constructing a self-organizing network of sensors using a secure protocol for direct data exchange between the nodes or through a third-party network. The obtained data can be aggregated and visualized at the dedicated server, indicating the geo-position of the device for collecting visualized data. Such online portal allows for quick response to critical changes in the selected parameters as well as in the data analysis for future prediction.

The main challenges of urban environmental monitoring are the deployment simplicity and flexibility in terms of the mesh network reconfiguration [49] as well as resistance to the “malicious” sensor connection [50]. The main problems include: (i) the difficulty of initializing a network with a large number of devices; (ii) connecting a new sensor to an existing network; (iii) network scalability; (iv) the ability to use a trusted sensor in a network location other than the legal installation place; and (v) the ability to detect a malicious device (sensor) presence.

In this paper, we propose an advanced protocol for the initial authentication and addition of sensor nodes to an existing distributed network. During the operation of the system, a secure data transfer protocol is implemented based on pairwise authentication of the sensors in terms of their location, which protects the system from the unauthorized introduction of a malicious sensor or a critical change in the location of the legally installed sensor, and also prevents from false information updates. The system aims to enable flexible and efficient support for potential sensor network topology dynamics. The resulting general overview of the environmental situation will allow responding to critical changes in the monitored parameters quickly. Nonetheless, a flexible network configuration feature aims to cover the monitored territories in order to obtain the most accurate and useful data that can be further used for the analysis of the urban situation and planning measures to improve it.

## 4. Security and Scalability for Environmental Monitoring Sensor Networks

Today, there are many critical security issues in the data transmission and processing in the scope of dynamic sensor networks with variable topology [51]. In particular, the critical problem is to provide a secure device “arrival” to the existing network since reconfiguration in a centralized manner may be challenging. In situations when a trusted authority is unavailable (for example, due to the connectivity issues), the operation of mutual device authentication becomes much more complicated [52].

This section is mainly focused on possible solutions for the sensor networks creation and providing support for secure mutual authentication of their sensors (nodes) that could be utilized for urban environmental monitoring.

**Figure 2:**
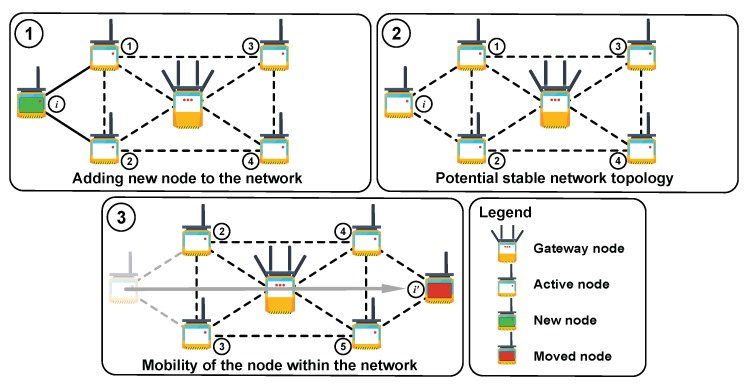
Monitoring systems operational states.

For our system, we assume that the network components are classified to only two groups, as shown in Figure 2:

- Gateway or Access Point (AP) is used for the end-node data aggregation. APs could also perform edge preprocessing of the incoming sensor data before the cloud delivery. Each data packet from each sensor node is encrypted using cloud public key to provide an additional level of the data integrity.
- Monitoring nodes are equipped with different sensing devices with the primary goal of collecting the specified environmental parameters, e.g., temperature, humidity, noise level, etc. The nodes could either connect directly to the AP or relay the data through the neighboring nodes to the AP in the ad hoc-like way.

The main abbreviations used in this work are given in Table 1 and the system operation could be divided into the following operational phases.

1. **Sensor initialization (addition)**: For example, a phase when a new node should be connected to any available node or AP in range (see Figure 2, Case 1). Assuming that both devices are operating in the same predefined way from the information security point of view, we consider two possible scenarios:
  - Simultaneous initialization of several sensors in one secure network. This situation is common for initial network deployment when a number of devices is more than two, k>2.
  - Adding a single new sensor to an existing secure sensor network.
2. **Stable sensor network operation**: In this scenario, sensors are neither added nor excluded from existing topology, and their logical position is static with respect to their neighbor nodes (see Figure 2, Case 2).
3. **Sensor migration**: In this scenario, the network faces the topology change (see Figure 2, Case 3) that could be caused by different factors:
  - Legally moved sensor is within the network segment with established pairwise relation;
  - Illegally moved sensor.
4. **Sensor removal**: In this scenario, two possible scenarios may be present:
  - Removed sensor is excluded from a particular secure network and could be used in the future only through new node initialization procedure.
  - Removed sensor is migrated to another segment of an existing network without reinitialization.

After careful evaluation of each of the mentioned scenarios, we decided to use the master key of sensor network [53,54] for initial authentication. At the first step of the sensor network initialization, it is necessary to provide mutual authentication for the single network segment. The segment is specified by the radio link range of the desired technology. For the sensor mutual authentication, we utilize the Lightweight Extensible Authentication Protocol (LEAP) -like protocol [55]. The main difference between common mutual authentication protocols for sensor networks on the stage of initialization is the level of master key protection on the next steps of the network life cycle:

1. The master key used on the initialization step is not removed and is kept in the so-called tamper resistance memory of the node [56]. This approach allows us to change the configuration of the network by simple displacement of the earlier installed node from one segment of the secure network to another (see Figure 2, Case 3). The displaced node can then authenticate with any other neighboring node in the same network if the nodes have the same master key. However, this feature becomes a disadvantage in the case it is necessary to prevent illegal movement (for example, if there is a need to be aware of the actual location of each node [57]). In this case, we should utilize an additional user authentication protocol for the system operator, which is required to make legal replacement of the active node, i.e., only the authenticated user should have an opportunity to move the sensor from one segment of the secure network to another. Any unauthorized movement should be prohibited.
2. The master key used at the step of initialization is destroyed after predefined time calculated from the moment when the initialization step was completed [55]. This scenario strongly limits the possibility of previously installed sensor movement from the initial sensor network segment to another part of the same network. This feature of the protocol allows obtaining a rather stable structure of the network. In this case, the probability of getting false information from the nodes is significantly reduced due to the location change.

Evidently, the second protocol is preferred in real-life dynamics of urban monitoring purposes. This protocol could be described as follows.

### 4.1. First Initialization of Several Sensors for New Secure Sensor Network

- Initially, the master key MK is defined for a new secure network. Each node *i* has its own unique identification number $ID_{i}$, $ID_{i}>ID_{j}$ for i>j. Next, we define one-way function—H(∗).
- During the initial initialization, nodes can only exchange data in wireless link range, as depicted in Figure 2 (Case 2). Here, sensors 1, 2 and 3 exchange their unique IDs $ID_{1},ID_{2}$, and $ID_{3}$.
- Each of the nodes utilizes the information about unique IDs of other sensors and the master key MK to calculate pair-wise keys for mutual authentication. For example, sensor 1 calculates pair keys for sensors 2 and 3 as:
  (1) $$\begin{array}{cc} \; & K_{1,2}=H(ID_{1}||ID_{2}||MK), \end{array}$$
  (2) $$\begin{array}{cc} \; & K_{1,3}=H(ID_{1}||ID_{3}||MK). \end{array}$$
  where x||y stands for the concatenation.
  Consequentially, sensors 2 and 3 also calculate the same pair-wise keys for the sensor 1:
  (3) $$\begin{array}{cc} \; & K_{2,1}=H(ID_{1}||ID_{2}\left||MK\right)=K_{1,2}, \end{array}$$
  (4) $$\begin{array}{cc} \; & K_{3,1}=H(ID_{1}||ID_{3}\left||MK\right)=K_{1,3}. \end{array}$$
- To provide the scalability, each sensor also calculates auxiliary key $K_{i,i}=H(ID_{i}\left||MK\right)$ for adding new sensors in the future.
- Each sensor removes its master key MK after predefined interval $T_{rm}$ from the first initialization process. This way, sensor 1 in Figure 2 (Case 2) would have the same information $\left\{K_{1,1},K_{1,2},K_{1,3}\right\}$ after the end of the initialization phase.

Generally, after deleting the master key from the memory of the node, secure communications would only be available with ones that have already established the pairwise keys at the initialization step of the protocol. However, each node should have the possibility to connect with new nodes for better system scalability. Each new node at the initialization step has a stored predefined MK—the node has a possibility to calculate $K_{i,i}$ as a pairwise key with already known node with $ID_{i}$ as $K_{i,i}=H(ID_{i}\left||MK\right)$.

### 4.2. Stable Sensor Network Operation

During the normal operation, nodes utilize pair keys that they have obtained during the first initialization for mutual authentication and generation of the session key. For example, sensors $ID_{1}$ and $ID_{2}$ use pair-wise keys $K_{1,2}$ and $K_{2,1}$ consequently.

### 4.3. Adding New Sensor to Existing Secure Sensor Network

According to Figure 2 (Case 2), a new $ID_{i}$ sensor appears in the range of sensors 1 and 2 of the existing network.

The new *i*th sensor should generate pair-wise keys for neighbor sensors 1 and 2 using master key MK (preinstalled earlier), and calculate new pair keys $K_{i,1}=H(ID_{1}\left||MK\right)=K_{1,1}$ and $K_{i,2}\;=\;H(ID_{2}||MK)\;=\;K_{2,2}$ to establish a connection with sensors 1 and 2. In this case, new node is treated as one legally added to the network.

On the next step, *i*th sensor should delete its master key MK. A new node should create a new auxiliary key $K_{i,i}$ before the master key removal. As a result, the new added node will store the key sequence $\left\{K_{i,i},K_{i,1},K_{i,2}\right\}$ after the initialization process.

### 4.4. Legal Sensor Moving to Another Secure Sensor Network Segment of Existing Network

We also consider the case of the sensor node migration from one network segment to another. For this scenario, we define the network segment $S_{i}$ as a subset of nodes $J(S_{i})$ that have previously established pairwise keys with this $ID_{i}$ node, i.e., $K_{i,j}=H(ID_{i}||ID_{j}\left||MK\right)$ or $K_{i,j}=H(ID_{i}\left||MK\right)$, $j\;\in \;J(S_{i})$.

Indeed, segment $S_{k}$ for a selected node $ID_{k}$ could also have some nodes from $S_{i}$, which is defined by the network topology. In the case node $ID_{i}$ is moved from $S_{i}$ to $S_{k}$, it will remain connected to nodes that are a part of both subsets $S_{k}\cap S_{i}$ and, therefore, existing pairwise keys could be used. In the case $S_{k}$ does not involve any nodes from $S_{i}$, the reinitialization of the node would be required. Fortunately, if the node $ID_{i}$ is moved back next to any known ones from $S_{i}$, it can have an opportunity to reinitialize the connectivity automatically.

### 4.5. Illegal Sensor Moving to Another Secure Sensor Network Segment of Existing Network

In the case of illegal sensor movement from $S_{i}$, e.g., without the master key MK updates (see Figure 2, Case 3), the process of mutual authentication will fail. This authentication failure will occur because the pair-wise key generated on the initialization step could not be used for any (new) neighbor sensors of a new segment due to the unique properties of the pairwise keys (similar to the legal movement procedure). This property of the authentication protocol decreases the probability of receiving incorrect data when the location of the node changes illegally.

## 5. Selected Numerical Results

The usage of routing and secure pairwise authentication protocols for legal network sensors [58] allowed us to cover a large part of territory without additional APs and by using already existing infrastructure for data aggregation, which potentially decreases the operational cost of the system. On the other hand, if we consider a farm monitoring IoT scenario, there is an open task to evaluate the required density of relatively cheap (compared to the AP price) sensors with respect to both coverage area and reliability.

In the simplest scenario, we may analyze the system from the network node density perspective. In particular, we focus on the scenario when the goal is to minimize the number of nodes while maintaining a high level of mesh reliability. We assume that the network segment has around one public transport stop equipped with the city public network AP per 400 m, which corresponds to the suboptimal traffic stops distance in the urban scenario for Europe [59]. At the same time, the node placement was selected to be on the lighting poles, generally separated by approximately 10 m in urban areas [60]. Therefore, the maximum number of potential placement locations is 39 between each pair of public transport stops, and thus a maximum number of potential hops in our mesh networks equals 40. In the worst scenario, some mesh network segments could be isolated if the connectivity to any AP is not available, which may be a result of inefficient nodes placement, e.g., when any node has only two links to its neighbors. By increasing the number of nodes, the overall reliability will grow along with the network cost.

In this work, we vary the availability of the sensor node for the lifetime of 10 years, which is suitable for environmental and urban monitoring [61,62]. As for the selected technology, the practical range is set to be 50 m [63].

**Figure 3:**
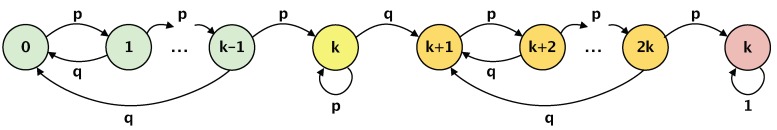
Markov chain utilized for the network segment analysis.

**Figure 4:**
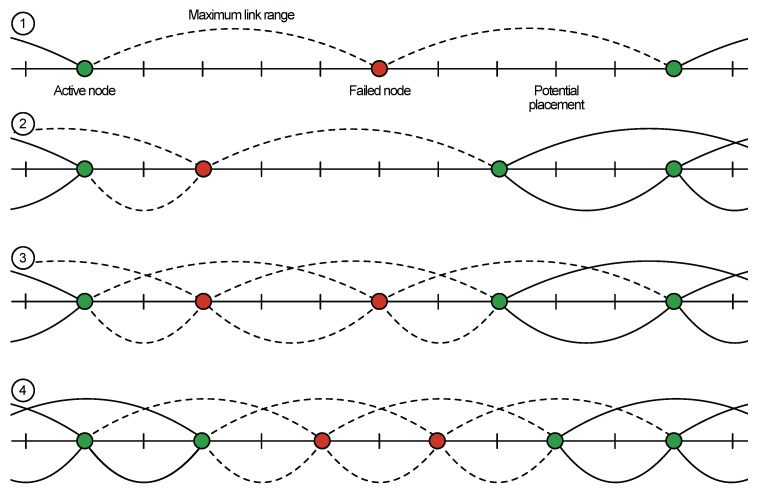
Potential node placement strategies: solid line, active link; dashed line, disrupted link.

We developed a Markov chain model (see Figure 3), describing the failure process of a series of sensor nodes with *k* overlapping connections shown in Figure 4. If a node has failed (with probability *p*), we make a transition to the right on the Markov chain, but if the node has not failed (with probability q=1-p), we make the transition to the left. To isolate a group of nodes in the presence of “k-extra” connections, *k* consecutive nodes must fail on both sides of a group. It can be represented as a stochastic counting process that moves to the next state if it encounters a failed node and returns to the initial state if it encounters a working node.

The Markov chain with the state space S={0,1,⋯,2k+1} has four communicating classes: 0,1,⋯,k-1,k,k+1,⋯,2k,2k+1. The first class represents the states in which we encountered less than *k* failed nodes in a row. Once we encountered ≥k failed nodes, we make a transition to the second class and stay in this class if we encounter more consecutive failed nodes. Once we encounter at least one working node (after a breaking sequence), we move to the third class. The fourth class is a final absorbing state, which is reached if we encounter ≥k failed nodes in a row for the second time. In summary, Class 1 represents the situation when we have a connection to both gateways. Classes 2 and 3 correspond to situations when a connection is lost to one of the gateways. Class 4 is the absorbing state 2k+1, which depicts the situations when we lose connection to both of the gateways. The transition probability matrix is given in Table 2, where *p* is the sensor failure probability and q=1-p. To find the probability of isolation of a group of nodes in a series of nodes of length *n*, we must find the *n*-step transition probability $P_{0,2k+1}^{\left(n\right)}$ from state 0 to state 2k+1.

**Figure 5:**
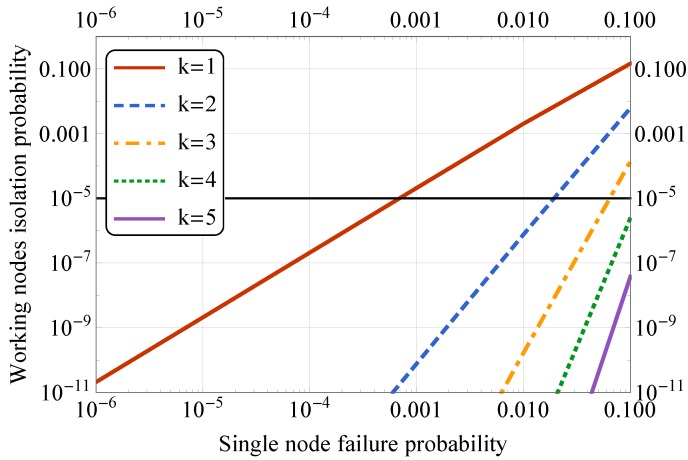
Effect of node placement density: between traffic stops.

In the first scenario, we focus on the mesh operation between two public transport stops, and the results are given in Figure 5. Here, both axes have a logarithmic scale. Here, the horizontal black line represents the overall system reliability equal to 99.999%, and we vary the probability of a single node to fail, thus, eliminating at least two links between the neighbors. By increasing *k* value, we introduce a higher number of additional links, as shown in Figure 4, which decreases the probability of the network segment separation. Following the overall reliability requirement, we can conclude that having k=3 almost reaches the required threshold and thus could be used for the actual system deployment. Therefore, the sensors could be placed at three out of five lighting poles for the node reliability of 99% per 10 years operational time.

**Figure 6:**
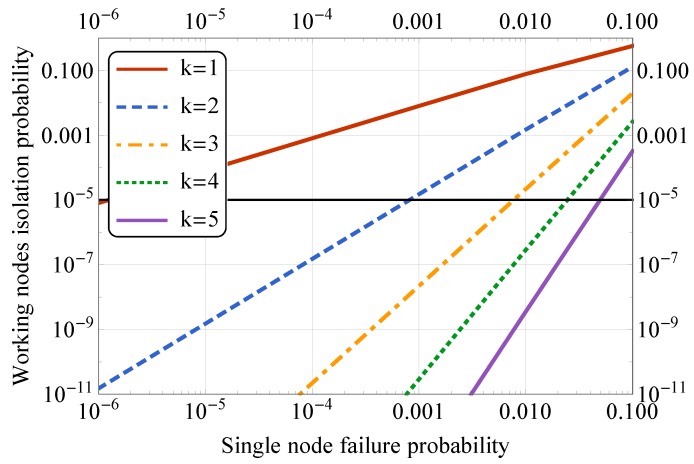
Effect of node placement density: edge operation.

For edge operation, i.e., when there is just one public transport stop available, we reduce the Markov chain by accumulating Classes 2–4 into a single absorbing state *k*, similar to state 2k+1 in a nonreduced case. The corresponding results are given in Figure 6. Evidently, the system reliability is much lower than compared to the first case, mainly due to a lower number of backup links available and a higher probability of the network separation in case of the close-to-AP node failure.

## 6. Prototyping Aspects

**Figure 7:**
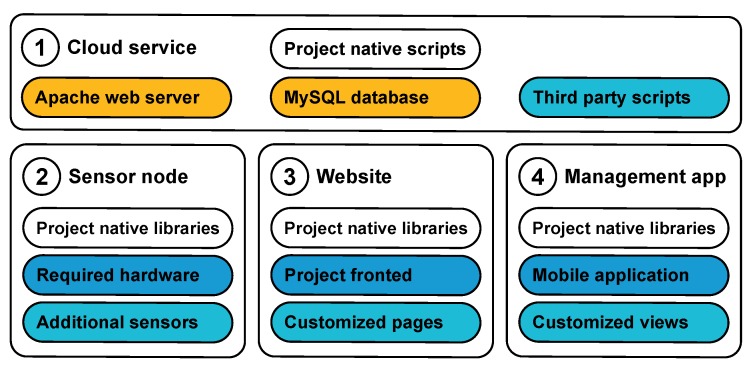
Main system components.

In this section, we describe our custom platform, which was developed aiming to improve the process of secure monitoring IoT system development ease and is based on the REpresentational State Transfer (REST) principle. Additionally, this platform improves the initialization process by the automated MK distribution and visualization of the node location on the map. The developed platform is a set of components allowing to build IoT solutions based on Atmel ATmega328P controller [64] equipped with wireless ESP8266 module [65]. The primary platform segments are: (i) firmware (binary image for ESP8266 chip); (ii) Android software (Java libraries and sample applications); (iii) web software (JavaScript library and sample pages); (iv) server-side services (user interface, data processing scripts, and database access scripts); and (v) database (MySQL). Their relations are highlighted with the same colors in Figure 7.

The primary goal of the platform was to handle issues related to security, connectivity, and access management, while the developer only needs to design the device and customize the data processing. In particular, the platform is prepared to be transparent for developers to perform the following:

1. Initialization “duckling” of the devices [66] by using any available wireless technology of the user smartphone, i.e., Bluetooth, WiFi, or NFC [67];
2. Access sharing;
3. Routing between devices;
4. Remote access; and
5. Setting up the network credentials, and other tasks.

The platform allows rapid development of the user application using Java library based on the following list of actions:

- To register in the cloud and generate its encryption key. In this case, the generated encryption key is stored only on the user smartphone but could be sent to the cloud.
- To perform node initialization.
- To interact with already initialized devices directly when they are in the communication range of the selected wireless technology.
- To specify access credentials of known APs and distribute those to all related devices.
- To interact with the devices via the infrastructure network. In this case, all transferred data are protected with end-to-end encryption between the smartphone and the node.

When initialized, ESP8266 can be accessed by Universal Asynchronous Receiver/ Transmitter (UART) protocol, e.g., it could be used to securely send/receive arbitrary JSON-packed data to/from server or smartphone.

**Figure 8:**
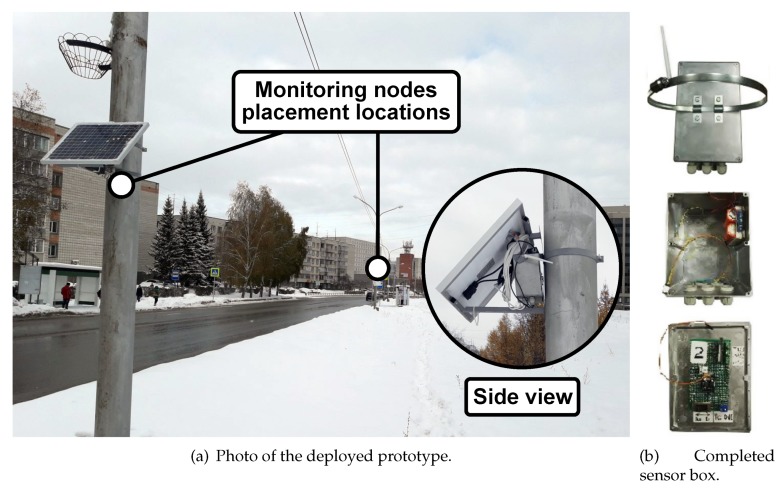
Trial-related photos.

According to the proposed platform and the above described protocols, we developed an urban monitoring system prototype based on ESP8266. Our nodes are currently equipped with the following sensors: CO${}_{2}$, radiation, and noise level. The deployment took place in Novosibirsk’s satellite city Koltsovo, Russia, and currently consists of seven monitoring devices. A photo of the deployed system is given in Figure 8. The complete device fulfills the requirements of IP 65. More technical details on the developed system are given in Table 3. In this project, we equipped our sensor nodes with three potential energy sources: (i) battery; (ii) solar panel; and (iii) wired power supply. The selection was made according to the need for autonomous operation and resistance to potential blackouts. Overall, the placement of the nodes on the lighting poles provides access not only to the powerline but allows for the utilization of energy harvesting technologies [68,69] that may be added to our project in the future.

For ease of use, we developed a custom monitoring data representation. The visualization side is a software module written in Hypertext Preprocessor (PHP) that generates a Hypertext Markup Language (HTML) views. Each view provides the user with an intuitive representation of the monitoring data received from the database after required Cloud processing. The information on the HTML page is updated via asynchronous requests. Measurements visualization, represented by the corresponding graphs, is carried out using the FlotJS library [71]. A sensor location map is generated using Yandex Maps API 2.0 [72], and the information about the location of each sensor is determined based on its initial placement.

The system is composed of two modules responsible for: (i) data analysis; and (ii) CU. The CU allows to modify the operating mode of each node (simplex, half-duplex, and duplex) remotely and provides the legal user with a mobile application for initializing sensors.

**Figure 9:**
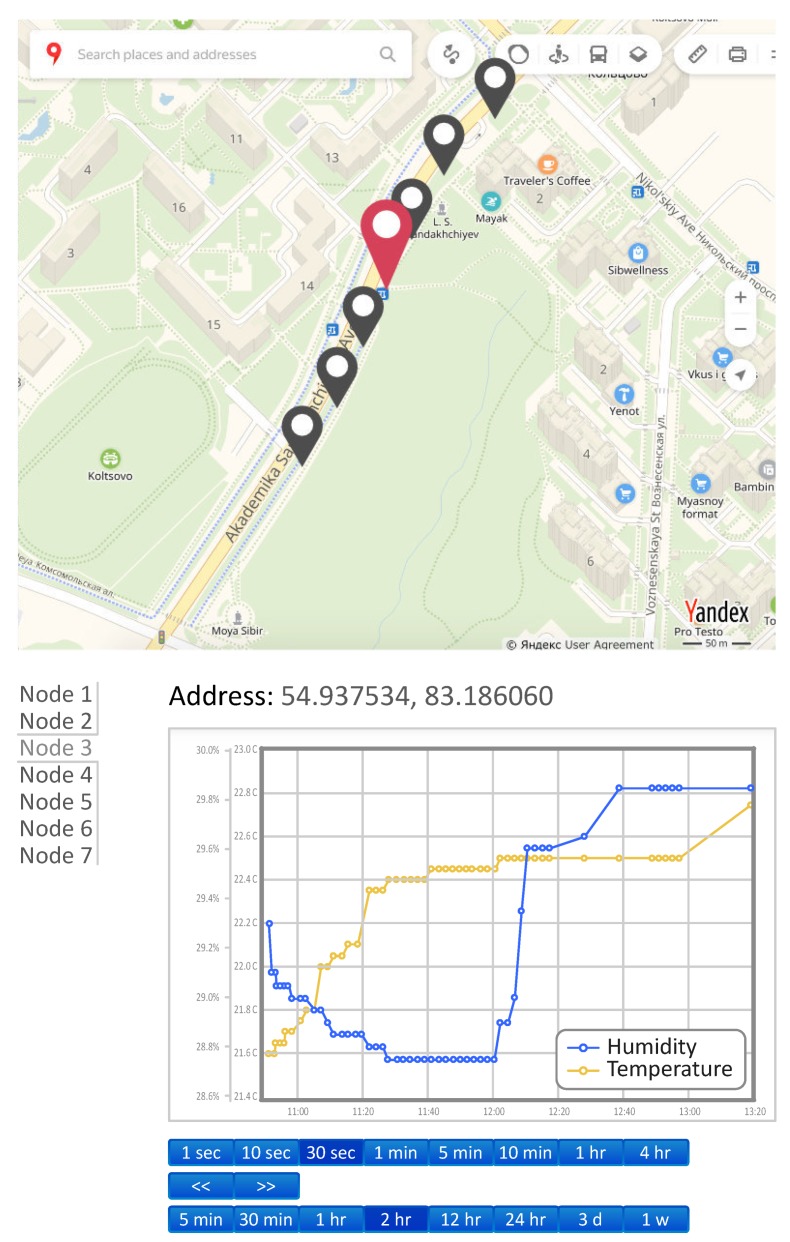
Application interface: Collected humidity and temperature view.

The overview of the user Dashboard view is given in Figure 9. The dashboard is a web page visually divided into three parts. At the top is a map with markers indicating the location of available sensors. A list of sensors is located on the left side, and on the right is the data area of the selected sensor. The selection of the sensor could be made either with the map or the list.

After the selection, the panel shows each sensor’s location along with the monitored environmental data visualized in the plot. The axes are scaled automatically according to the data received. The representation could be changed based on the control buttons located below the plot. For example, there is a possibility for scrolling the graph to overview previous results, selection of the displayed data range, and control of the measuring interval of the sensor in half-duplex mode. From the developed authentication methodology perspective, we tested all the cases listed in Section 4 during the deployment successfully.

## 7. Conclusions

Climate change brings the problem of environmental monitoring to an entirely new level, especially for urban scenarios. In particular, the leading cause of indoor air pollution is inefficient fuel combustion from rudimentary technologies used for cooking, heating, and lighting in addition to complex traffic conditions in metropolitans. All of the above require practical and flexible enablers for monitoring the emission levels, temperature, and other factors affecting citizens’ lives.

In this work, we first discussed a pairwise key-based authentication methodology followed by the prototype of the secure urban environmental monitoring system and the executed field trial. Despite conventional sensor network goals, the system allows protecting a sensor network from unauthorized topology changes, keeping the properties of scalability and security from a communications perspective. The platform was developed to enable efficient and fast network initialization, received information processing, and handling potential topology changes. The use of mutual authentication protocol, together with our platform, allowed us to build an efficient, safe, and easily scalable sensor network to collect and process environmental information. The expertise collected during this system prototyping would be further used for the DLT design principles formulation.

Concluding, the developed system received positive feedback from the customer (DELL) and the research community during the IoT Summit Siberia, where the solution was presented to the broad public. The mayor of the city also provided his vision on how to further utilize the system for environmental and Smart City purposes. The developed system could also be efficiently utilized in farm and suburban scenarios where the connectivity to the gateway access point is relatively close to any segment of the mesh network.

## Figures and Tables

**Table 1:** Main notations used in the paper.

Notation	Description
*k*	Number of sensors
i,j,l,n	Indexes
MK	Master Key
$ID_{i}$	*i*th sensor node unique identifier
H(∗)	One-way function
$K_{i,j}$	Auxiliary key for *i*th and *j*th nodes
$T_{rm}$	MK lifetime period after the initialization phase
$S_{i}$	Subset of nodes that have pairwise connection with *i*th node
$J(S_{i})$	Number of nodes in $S_{i}$
*p*	Single node failure probability (q=1-p)
*S*	State space of the Markov chain
$P_{i,j}$	Transition probability from state *i* to state *j*

**Table 2:** Transition probability matrix.

State	0	1	⋯	*k*− 1	k	*k* + 1	⋯	2*k*	2*k* + 1
0	q	p	0	⋯	⋯	⋯	⋯	⋯	0
1	q	0	p	0	⋯	⋯	⋯	⋯	0
⋮	⋮			⋱					⋮
*k*− 1	q	0	⋯	0	p	0	⋯	⋯	0
*k*	0	⋯	⋯	0	p	q	0	⋯	0
*k* + 1	0	⋯	⋯	⋯	0	q	p	0	0
⋮	⋮					⋮		⋱	⋮
2*k*	0	⋯	⋯	⋯	0	q	0	0	p
2*k* + 1	0	⋯	⋯	⋯	⋯	0	⋯	0	1

**Table 3:** Main components of the node.

Component	Type	Description
Atmel ATmega328P	Data processing and control	Micro-controller is dedicated to the system operation, which holds the functionality of the data processing unit (DPU) and control unit (CU) [64].
Data Processing Unit	Data processing and control	DPU is implemented in ATmega328P and performs the functions of preprocessing information received from sensors for secure and reliable transmission to the server unit. Data pre-processing is carried out in accordance with the previously developed and used Galouis platform.
Control Unit	Data processing and control	CU is implemented in ATmega328P and ensures the operation of the radio module and the DPU, determining their operation in various modes in accordance with the Galois platform used. Besides, CU regulates the mode of operation of the sensors, ensuring efficient energy consumption in the respective modes of the system (simplex, half-duplex, and full-duplex), and also allows the interaction through the radio module with the mobile device during the initialization of the sensor and the end of its operation.
ESP8266 radio module	Communications	Provides data transfer via IEEE 802.11n protocol [65]. The radio module receives data from DPU according to the control commands from the CU and transfers it to the networking part of the system or the nearest sensor located in its communications range. Note, in the duplex mode of operation, the radio module relays the data received from the sensors located in its coverage area according to the commands received from the control unit.
Power Control Unit (PCU)	Power supply	Provides safe switching between available power sources in order to realize the uninterrupted power supply of the sensor, regardless of weather conditions and the state of available power sources. As a baseline element, the system utilizes the SII-8205A board [70].
Battery	Power supply	Li-ion, 6800 mAh, 3.7 V.
Solar panel	Power supply	45 W, 12 V (optional).
Power source	Power supply	12 V, 2 A (optional).

## Abbreviations

The following abbreviations are used in this manuscript:

AP	Access Point
API	Application Programming Interface
CPS	Cyber-Physical System
CU	Control Unit
DPU	Data Processing Unit
GIS	Geographical Information Systems
GUI	Graphical User Interface
HTML	Hypertext Markup Language
ID	Identifier
IEEE	Institute of Electrical and Electronics Engineers
IIoT	Industrial Internet of Things
IoT	Internet of Things
IP (Code)	International Protection Marking
JSON	JavaScript Object Notation
LEAP	Lightweight Extensible Authentication Protocol
M2M	Machine-to-Machine Communications
MK	Master Key
NASA	The National Aeronautics and Space Administration
NFC	Near Field Communications
PCU	Power Control Unit
PHP	Hypertext Preprocessor
PKI	Public Key Infrastructure
REST	Representational State Transfer
RFID	Radio-frequency Identification
SQL	Structured Query Language
UART	Universal Asynchronous Receiver/Transmitter
UWB	Ultra-wideband Radio Technology
WiFi	Wireless Fidelity
WSN	Wireless Sensor Network
